# Differentiating between benign and malignant breast lesions using dual-energy CT-based model: development and validation

**DOI:** 10.1186/s13244-024-01752-2

**Published:** 2024-07-10

**Authors:** Han Xia, Yueyue Chen, Ayong Cao, Yu Wang, Xiaoyan Huang, Shengjian Zhang, Yajia Gu

**Affiliations:** 1 Department of Radiology, Fudan University Shanghai Cancer Center and Department of Oncology, Shanghai Medical College, Fudan University, Shanghai, 200032 China; 2 Key Laboratory of Breast Cancer in Shanghai, Department of Breast Surgery, Fudan University Shanghai Cancer Center and Department of Oncology, Shanghai Medical College, Fudan University, Shanghai, 200032 China; 3 Clinical and Technical Support, Philips Healthcare, Shanghai, 200072 China

**Keywords:** Breast neoplasms, Diagnostic imaging, Dual-energy computed tomography, Logistic models, Quantitative parameters

## Abstract

**Objectives:**

To develop and validate a dual-energy CT (DECT)-based model for noninvasively differentiating between benign and malignant breast lesions detected on DECT.

**Materials and methods:**

This study prospectively enrolled patients with suspected breast cancer who underwent dual-phase contrast-enhanced DECT from July 2022 to July 2023. Breast lesions were randomly divided into the training and test cohorts at a ratio of 7:3. Clinical characteristics, DECT-based morphological features, and DECT quantitative parameters were collected. Univariate analyses and multivariate logistic regression were performed to determine independent predictors of benign and malignant breast lesions. An individualized model was constructed. Receiver operating characteristic (ROC) curve analysis was performed to evaluate the diagnostic ability of the model, whose calibration and clinical usefulness were assessed by calibration curve and decision curve analysis.

**Results:**

This study included 200 patients (mean age, 49.9 ± 11.9 years; age range, 22–83 years) with 222 breast lesions. Age, lesion shape, and the effective atomic number (Zeff) in the venous phase were significant independent predictors of breast lesions (all *p* < 0.05). The discriminative power of the model incorporating these three factors was high, with AUCs of 0.844 (95%CI 0.764–0.925) and 0.791 (95% CI 0.647–0.935) in the training and test cohorts, respectively. The constructed model showed a preferable fitting (all *p* > 0.05 by the Hosmer-Lemeshow test) and provided enhanced net benefits than simple default strategies within a wide range of threshold probabilities in both cohorts.

**Conclusion:**

The DECT-based model showed a favorable diagnostic performance for noninvasive differentiation between benign and malignant breast lesions detected on DECT.

**Critical relevance statement:**

The combination of clinical and morphological characteristics and DECT-derived parameter have the potential to identify benign and malignant breast lesions and it may be useful for incidental breast lesions on DECT to decide if further work-up is needed.

**Key Points:**

- It is important to characterize incidental breast lesions on DECT for patient management.
- DECT-based model can differentiate benign and malignant breast lesions with good performance.
- DECT-based model is a potential tool for distinguishing breast lesions detected on DECT.

**Graphical Abstract:**

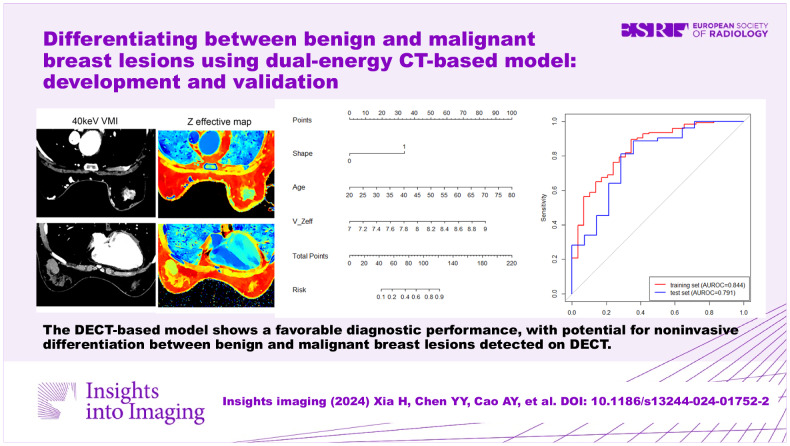

## Introduction

Breast cancer is now the most common cancer and the leading cause of cancer-related deaths among women [1]. Despite not being a conventional method for assessing breast lesions, the growing prevalence and utilization of computed tomography have revealed that incidental breast lesions are detected in a range of 0.3% to 7.63% of cases [2–4]. Furthermore, 31.0% to 70.0% of these incidental breast lesions have been found to be malignant upon pathological findings [5, 6]. Hence, incidental breast lesions on CT represent a clinical dilemma for the general radiologist to decide if further work-up is needed.

Dual-energy CT is a technique that collects two sets of raw data of high and low energies [7]. A wide range of virtual monoenergetic images (VMIs) and material decomposition images may be obtained and reconstructed to increase contrast enhancement between soft tissues and for material separation. DECT has exhibited potential clinical applications in tumor imaging [8–12]. Several studies have used DECT to determine optimal energies for VMIs to display breast lesions [13–15], differential diagnosis of benign and malignant breast lesions [16–18], the assessment of lymph node metastasis [19–21], the detection of distant metastasis in breast cancer [22, 23], and the prediction of the status of immunohistochemical biomarkers of breast cancer [24–26]. Considering that DECT is now routinely used for clinical examinations and offers relatively high tissue resolution, it might be helpful in characterizing multifocal or multicentric breast lesions during the systemic staging of breast cancers, potentially reducing the need for MRI. Furthermore, it could be beneficial to differentiate incidental breast lesions on DECT to reduce the number of unnecessary referrals to breast units. Previous reports [16–18, 27] have indicated that DECT quantitative parameters, including iodine concentration (IC), effective atomic number (Zeff), and the slope of the curve (λ_HU_), have good diagnostic values in discriminating between benign and malignant breast lesions. However, no studies have considered the clinical and imaging characteristics for differential diagnosis. It is well known that patients with benign and malignant breast lesions have differences in clinical features [28] and imaging manifestations [5, 6, 29–31], and considering this information may be helpful in differential diagnosis and would better align with clinical practice.

We hypothesized that DECT quantitative parameters combined with clinical and morphological features could be useful for the characterization of benign and malignant breast lesions. The aim of this study was to develop and validate a DECT-based model for differentiating between benign and malignant breast lesions detected on DECT to aid in further work-up.

## Materials and methods

This prospective, single-center study was approved by the ethics committee of our institution, and all individuals provided signed informed consent.

### Patient cohort

**Fig. 1:**
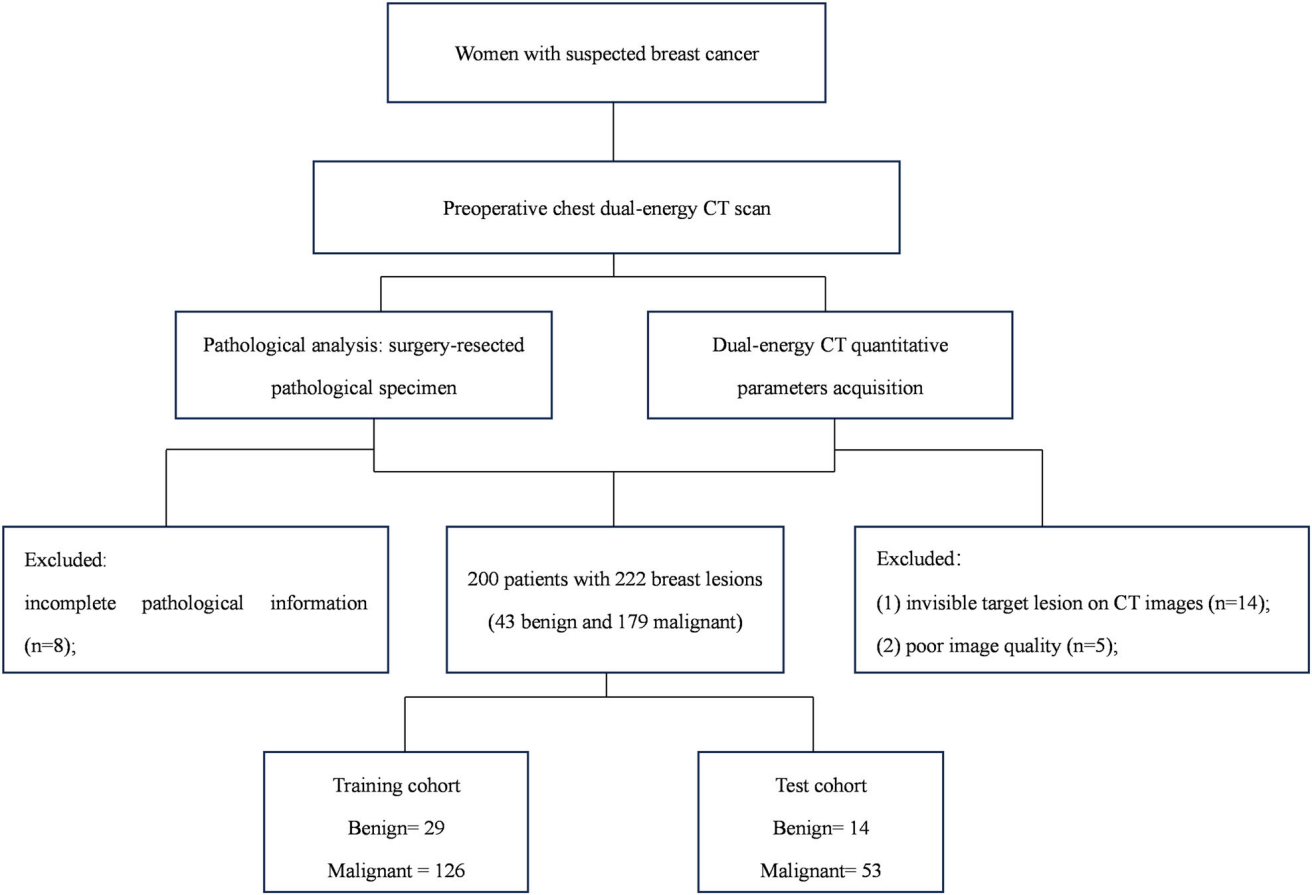
Study flowchart

Our study consecutively enrolled patients with suspected breast cancer who underwent chest dual-phase contrast-enhanced DECT scans before surgeries for evaluation of the status of mediastinal and axillary lymph nodes or underlying lung neoplasms from July 2022 to July 2023 according to our local protocol. Inclusion criteria were: (1) BI-RADS 4 A/4B/4 C or 5 breast lesions detected by ultrasound or mammography; (2) no previous exposure to chemotherapy or radiotherapy of the breast; (3) no history of iodine allergy or renal insufficiency (estimated glomerular filtration rate ≤ 30 mL/min). Exclusion criteria were: (1) incomplete pathological information, (2) invisible target lesions on DECT images, and (3) poor image quality caused by severe metal artifacts. Eligible breast lesions were included and randomly divided into training and test cohorts at a ratio of 7:3. A study flowchart is shown in Fig. 1.

### Clinical and morphological characteristics

According to previous studies [32, 33], the odds ratio (OR) for breast cancer increased with increasing FGT and BPE. Clinical features and morphological characteristics from DECT images were collected, including age, family history, menopausal status, symptoms, lesion location, fibroglandular tissue (FGT), background parenchymal enhancement (BPE), lesion shape, margin, lesion enhancement, inner enhancement of lesions, and maximum lesion diameter. Two radiologists with two years of experience in breast imaging who were blinded to pathological findings evaluated DECT-based morphological characteristics in consensus, resolving any discrepancies through consultation with a senior radiologist with 20 years of experience in breast imaging. The assessment of imaging morphological features was based on the MRI-BI-RADS lexicon [34] and a previous study [30]. On DECT images, breasts with almost entirely fat or scattered fibroglandular tissue (BI-RADS A or B) were classified as non-dense FGT, while breasts with heterogeneous or extremely dense fibroglandular tissue (BI-RADS C or D) were classified as dense FGT. Minimal and mild BPE were categorized as mild, while moderate and marked BPE were categorized as moderate/marked. Oval and round masses were considered regular shapes, otherwise, they were considered irregular in lesion shape. Irregular or spiculated margins were non-circumscribed. Non-mass enhancement (NME) included focal, linear, segmental, regional, multiple regions, and diffuse enhancement. Rim enhancement for mass enhancement and clumped or clustered ring enhancement for NME were classified as inner heterogeneous enhancement. Histopathological data of all lesions were extracted from the pathological reports in the electronic medical records system.

### DECT image acquisition

All participants underwent chest contrast-enhanced CT with a 128-row spectral dual-layer detector CT scanner (IQon Spectral CT, Philips Health Systems) in the prone position for proper spreading of breast tissues to facilitate the visualization of any abnormalities. The detailed DECT protocol is provided in Appendix Table E1 (electronic supplementary material). An iodinated contrast agent (Ultravist 370, Bayer Schering Pharma) at 1.5 mL/kg was injected via the antecubital vein at 2–3 mL/s in each enrolled subject, followed by administration of 30 mL saline at the same rate. Arterial phase scanning was initiated using a bolus-tracking method with a 100 Hounsfield unit (HU) threshold in the descending aorta and an additional delay of 10 s. The venous phase scan was started 25 s after the end of the arterial phase scan.

We recorded the CT dose index volume (CTDIvol) and the dose length product (DLP) for each patient, and by multiplying the DLP by a conversion factor (k = 0.014 mSv/mGy · cm), the effective radiation dose was determined [35].

### DECT quantitative parameters

DECT images were automatically reconstructed on a dedicated workstation (IntelliSpace Portal 10.0, Philips Healthcare). DECT quantitative parameters were measured by two radiologists with 2 years of experience in breast imaging in consensus, who were blinded to pathological findings, and any disagreements were resolved by consulting a senior radiologist with 20 years of experience in breast imaging. Circular regions of interest (ROIs) were placed on axial slices showing the maximum dimension of each breast lesion. ROIs were adjusted as large as possible on the lesions while excluding obvious necrosis, calcifications, and major vessels. Similar-sized ROIs were also placed on adjacent upper and lower slices, as well as on normal breast parenchyma and the aorta for background comparison. An example of ROI placement is illustrated in Appendix Figure E1. For each lesion, ROIs were kept consistent on both arterial- and venous-phase DECT images with the copy-and-paste function in the workstation. All measurements were performed on three slices and then averaged.

The DECT quantitative parameters of attenuation (HU) on conventional images and 40-keV VMIs, IC (mg/mL), Zeff (absolute numbers), and λ_HU_ in the arterial and venous phase were measured. The IC and Zeff of breast lesions were normalized by dividing them by the corresponding values for the aorta or normal breast parenchyma (nIC and nZeff), respectively. The nIC, nZeff, and λ_HU_ of breast lesions were determined as follows:

$${nIC}=\frac{{{IC}}_{{breast\; lesion}}\,\left({mg}/{mL}\right)}{{{IC}}_{{aorta}/{normal\; glandular\; tissue}}\,\left({mg}/{mL}\right)}$$

$$n{Zeff}=\frac{{{Zeff}}_{{breast\; lesion}}\,}{{{Zeff}}_{{aorta}/{normal\; glandular\; tissue}}\,}$$

$${\lambda }_{{HU}}=\frac{{{HU}}_{40{keV}}-{{HU}}_{70{keV}}\,}{\left(70-40\right){keV}\,}$$

### DECT-based model construction and evaluation

The clinical characteristics, DECT morphological indexes, and DECT quantitative parameters of breast lesions were compared by univariable analysis based on pathological findings in the training cohort. Only parameters with *p* < 0.05 in univariate analysis were selected as candidates for the subsequent multivariate logistic regression to identify independent predictors. According to the relevant independent predictors and respective regression coefficients, a DECT-based model was developed to differentiate between benign and malignant breast lesions detected on DECT.

The nomogram visualized the model to make it readable and operable. The diagnostic performance of the model was assessed by determining the area under the ROC curve (AUC) and its corresponding 95% confidence interval (CI), and the DeLong test was performed to compare AUCs in the training and test cohorts to assess the outfitting and robustness of the model. The sensitivity, specificity, and accuracy of the model in both cohorts were also determined. The Hosmer-Lemeshow test and calibration curves were utilized to evaluate the goodness of fit of the model. Furthermore, decision curve analysis was performed to determine the clinical utility of the model in both cohorts.

### Statistical analysis

A data analyst who was unaware of the parameters performed statistical analyses. Data normality was assessed using the Kolmogorov-Smirnov test. Normally distributed continuous variables were expressed as mean ± standard deviation and non-normally distributed variables as median and quartiles. Categorical variables were expressed as frequency and frequency distribution. In univariate analysis, both groups were compared by the independent t-test or Mann-Whitney U test for continuous variables and the chi-square test or Fisher’s exact test for categorical variables. Multivariate logistic regression analysis was conducted to identify independent factors differentiating between benign and malignant breast lesions. To obtain the optimal model, the R package function ‘glm()’ was used to select the model with the smallest Akaike’s Information Criteria and to remove any nonsignificant variables. The R software (version 4.2.1; R Foundation for Statistical Computing) was used for data analyses, and *p* < 0.05 indicated statistical significance.

## Results

### Clinical and morphology characteristics

This study enrolled 227 patients with suspected breast cancers. Patients were excluded due to incomplete pathological information (*n* = 8), invisible target lesions on DECT images (*n* = 14), and poor image quality caused by severe metal artifacts (*n* = 5). Finally, this study included 222 breast lesions in 200 patients (mean age, 49.9 ± 11.9 years; age range, 22–83 years). All breast lesions (100%) were diagnosed by surgical samples, and pathological findings revealed that 43 lesions were benign and 179 were malignant. The clinical characteristics and DECT-derived morphological features of the patients in the training and test cohorts are summarized in Table 1 and the pathological findings are shown in Appendix Table E2. No significant differences were found between the two cohorts in terms of age (50.1 ± 11.6 vs 49.3 ± 12.7, *p* = 0.63), family history (14.8% vs. 16.4%, *p* = 0.92), location (49% vs. 46.3%, *p* = 0.82), FGT (74.8% vs. 82.1%, *p* = 0.31), BPE (65.1% vs. 56.7%, *p* = 0.30), menopausal status (54.8% vs. 44.8%, *p* = 0.22), symptoms (60.6% vs 62.7%, *p* = 0.89), shape (31.0% vs. 35.8%, *p* = 0.58), margin (7.1% vs. 6.0%, *p* > 0.99), enhancement (74.8% vs. 79.1%, *p* = 0.61), inner enhancement (43.9% vs 50.7%, *p* = 0.43), and max diameter (2.52 ± 1.50 vs. 2.38 ± 1.17, *p* = 0.44).

**Table 1:** Clinical and morphological characteristics of benign and malignant breast lesions in the training and test cohorts

Variable	Training cohort				Test cohort			
	All (*n* = 155)	Malignant (*n* = 126)	Benign (*n* = 29)	*p*	All (*n* = 67)	Malignant (*n* = 53)	Benign (*n* = 14)	*p*
Age (y)	50.1 ± 11.6	52.0 ± 9.9	41.9 ± 14.6	0.001	49.3 ± 12.7	51.2 ± 11.3	41.9 ± 15.1	0.046
Family history				0.57				0.69
Present	23 (14.8)	20 (15.9)	3 (10.3)		11 (16.4)	8 (15.1)	3 (21.4)	
Absent	132 (85.2)	106 (84.1)	26 (89.7)		56 (83.6)	45 (84.9)	11 (78.6)	
Location				0.26				0.56
Left	76 (49.0)	65 (51.6)	11 (37.9)		31 (46.3)	26 (49.1)	5 (35.7)	
Right	79 (41.0)	61 (48.4)	18 (62.1)		36 (53.7)	27 (50.9)	9 (64.3)	
FGT				0.18				1.00
Dense	116 (74.8)	91 (72.2)	25 (86.2)		55 (82.1)	43 (81.1)	12 (85.7)	
Nondense	39 (25.2)	35 (27.8)	4 (13.8)		12 (17.9)	10 (18.9)	2 (14.3)	
BPE				0.06				0.79
Mild	101 (65.1)	87 (69.0)	14 (48.3)		38 (56.7)	31 (58.5)	7 (50.0)	
Moderate/marked	54 (34.9)	39 (31.0)	15 (51.7)		29 (43.2)	22 (41.5)	7 (50.0)	
Menopausal status				0.006				0.09
Premenopausal	85 (54.8)	62 (49.2)	23 (79.3)		30 (44.8)	27 (50.9)	3 (21.4)	
Postmenopausal	70 (45.2)	64 (50.8)	6 (20.7)		37 (55.2)	26 (49.1)	11 (78.6)	
Symptoms				0.65				0.008
Mass	94 (60.6)	78 (61.9)	16 (55.2)		42 (62.7)	38 (71.7)	4 (28.6)	
Others	61 (39.4)	48 (38.1)	13 (44.8)		25 (37.3)	15 (28.3)	10 (71.4)	
Shape				< 0.001				0.35
Regular	48 (31.0)	30 (23.8)	18 (62.1)		24 (35.8)	17 (32.1)	7 (50.0)	
Irregular	107 (69.0)	96 (76.2)	11 (37.9)		43 (64.1)	36 (67.9)	7 (50.0)	
Margin				0.006				0.03
Circumscribed	11 (7.1)	5 (4.0)	6 (20.7)		4 (6.0)	1 (1.9)	3 (21.4)	
Noncircumscribed	144 (92.9)	121 (96.0)	23 (79.3)		63 (94.0)	52 (98.1)	11 (78.6)	
Enhancement				1.00				1.00
Mass	116 (74.8)	94 (74.6)	22 (75.9)		53 (79.1)	42 (79.2)	11 (78.6)	
Nonmass	39 (25.1)	32 (25.4)	7 (24.1)		14 (20.9)	11 (20.8)	3 (21.4)	
Inner enhancement				0.12				0.15
Homogeneous	68 (43.9)	51 (40.5)	17 (58.6)		34 (50.7)	24 (45.3)	10 (71.4)	
Heterogeneous	87 (56.1)	75 (59.5)	12 (41.4)		33 (49.3)	29 (54.7)	4 (28.6)	
Max diameter	2.52 ± 1.50	2.56 ± 1.44	2.34 ± 1.75	0.056	2.38 ± 1.17	2.43 ± 1.04	2.18 ± 1.61	0.59

Quantitative variables are mean ± standard deviation. Categorical or qualitative variables were expressed as frequency and frequency distribution. Data in the brackets indicated the percentage of different clinical and imaging features in different cohorts

In the training cohort, univariate analysis showed that patients with malignant breast lesions were older (52.0 ± 9.9 vs. 41.9 ± 14.6, *p* = 0.001), more frequently postmenopausal (50.8% vs. 20.7%, *p* = 0.006), and exhibited more irregular shape (76.2% vs. 37.9%, *p* < 0.001) and noncircumscribed margins (96% vs. 79.3%, *p* = 0.006) compared with those with benign lesions.

The mean CTDIvol for each phase in this study was 6.40 ± 0.85 mGy. The DLP was 233.0 ± 11.31 mGy · cm, and the mean effective radiation dose was 3.26 ± 0.16 mSv.

### DECT quantitative parameters

Table 2 summarizes the DECT quantitative parameters of benign and malignant breast lesions in the training and test cohorts. In the arterial and venous phases, the attenuation on conventional images (50.2 ± 14.4 vs. 43.6 ± 12.7, *p* = 0.02 and 85.9 ± 19.8 vs. 70.9 ± 26.3, *p* = 0.007, respectively) and 40-keV VMIs (71.0 [52.9–95.4] vs. 52.7 [37.8–84.5], *p* = 0.04 and 177.8 ± 56.2 vs. 140.5 ± 74.1, *p* = 0.02), and in the venous phase, IC (1.58 ± 0.66 vs. 1.20 ± 0.84, *p* = 0.03), Zeff (8.14 ± 0.32 vs 7.92 ± 0.45, *p* = 0.02), nIC_lesion/normal glandular tissue_ (12.37[6.48–23.01] vs. 5.39 [3.29–14.37], *p* = 0.006), nZeff_lesion/normal glandular tissue_ (1.13 ± 0.05 vs. 1.09 ± 0.06, *p* = 0.01) and λ_HU_ (3.1 ± 1.31 vs. 2.31 ± 1.7, *p* = 0.03) were higher for malignant lesions than benign lesions in the training cohort. The diagnostic performances of these quantitative parameters in the training cohort are illustrated in Table 3. ROC curve analysis showed that these parameters had comparable diagnostic abilities, with a maximum AUC of up to 0.68.

**Table 2:** DECT quantitative parameters of benign and malignant breast lesions in the training and test cohorts

Variable	Training cohort				Testing cohort			
	All (*n* = 155)	Malignant (*n* = 126)	Benign (*n* = 29)	*p*	All (*n* = 67)	Malignant (*n* = 53)	Benign (*n* = 14)	*p*
Conventional attenuation (HU)								
In the arterial phase	48.9 ± 14.3	50.2 ± 14.4	43.6 ± 12.7	0.02	48.4 ± 11.3	49.8 ± 10.6	43.2 ± 12.6	0.09
In the venous phase	83.1 ± 21.8	85.9 ± 19.8	70.9 ± 26.3	0.007	83.9 ± 19.4	87.6 ± 17.6	70.2 ± 20.3	0.009
40-keV VMIs attenuation (HU)								
In the arterial phase	70.5 (48.0–92.7)	71.0 (52.9–95.4)	52.7 (37.8–84.5)	0.04	65.7 (48.4–87.1)	70.4 (54.3–87.9)	55.8 (42.4–61.5)	0.051
In the venous phase	170.9 ± 61.5	177.8 ± 56.2	140.5 ± 74.1	0.02	175.8 ± 50.4	184.9 ± 46.5	141.3 ± 51.2	0.009
IC (mg/mL)								
In the arterial phase	0.37 (0.12–0.65)	0.38 (0.14–0.65)	0.30 (0.05–0.62)	0.22	0.32 (0.13–0.52)	0.33 (0.21–0.5)	0.16 (0.06–0.54)	0.28
In the venous phase	1.51 ± 0.71	1.58 ± 0.66	1.20 ± 0.84	0.03	1.57 ± 0.57	1.66 ± 0.53	1.23 ± 0.59	0.02
nIC (ratio of lesion and aorta)								
In the arterial phase	0.04 (0.01–0.08)	0.05 (0.02–0.08)	0.04 (0.01–0.07)	0.20	0.04 (0.02–0.06)	0.04 (0.03–0.06)	0.02 (0.01–0.06)	0.22
In the venous phase	0.29 ± 0.14	0.31 ± 0.12	0.24 ± 0.18	0.07	0.29 (0.24–0.37)	0.3 (0.27–0.40)	0.26 (0.19–0.30)	0.04
nIC (ratio of lesion and normal parenchyma)								
In the arterial phase	3.00 (1.27–7.58)	3.23 (1.40–8.09)	2.31 (0.64–4.67)	0.12	3.00 (1.08–11.19)	3.35 (1.86–12.5)	2.05 (0.67–4.45)	0.12
In the venous phase	11.00 (5.30–22.48)	12.37 (6.48–23.01)	5.39 (3.29–14.37)	0.006	12.79 (5.80–19.59)	15.21 (8.15–21.08)	3.87 (2.02–6.54)	< 0.001
Zeff								
In the arterial phase	7.46 (7.30–7.66)	7.47 (7.32–7.66)	7.37 (7.26–7.62)	0.07	7.43 (7.31–7.58)	7.45 (7.33–7.58)	7.33 (7.23–7.46)	0.055
In the venous phase	8.1 ± 0.36	8.14 ± 0.32	7.92 ± 0.45	0.02	8.14 ± 0.28	8.19 ± 0.25	7.95 ± 0.3	0.01
nZeff (ratio of lesion and aorta)								
In the arterial phase	0.71 (0.68–0.73)	0.71 (0.68–0.73)	0.69 (0.66–0.73)	0.14	0.71 ± 0.03	0.71 ± 0.03	0.7 ± 0.03	0.63
In the venous phase	0.84 ± 0.04	0.85 ± 0.04	0.83 ± 0.05	0.08	0.85 (0.83–0.87)	0.85 (0.83–0.87)	0.84 (0.82–0.86)	0.08
nZeff (ratio of lesion and normal parenchyma)								
In the arterial phase	1.05 (1.02–1.07)	1.05 (1.02–1.07)	1.03 (1.01–1.06)	0.057	1.03 (1.02–1.07)	1.03 (1.02–1.07)	1.03 (1.01–1.05)	0.20
In the venous phase	1.12 ± 0.05	1.13 ± 0.05	1.09 ± 0.06	0.01	1.12 ± 0.05	1.14 ± 0.04	1.07 ± 0.05	0.001
λ_HU40-70 keV_ (HU/keV)								
In the arterial phase	0.63 (0.14–1.26)	0.69 (0.19–1.27)	0.33 (0–1.14)	0.054	0.55 (0.16–0.98)	0.62 (0.22–1.00)	0.21 (−0.09–0.62)	0.053
In the venous phase	2.95 ± 1.42	3.1 ± 1.31	2.31 ± 1.7	0.03	3.06 ± 1.13	3.26 ± 1.05	2.32 ± 1.13	0.01

Data are mean ± standard deviation or median and quartiles. *λ*_*HU40-70 keV*_ slope of the spectral Hounsfield unit curve between 40 and 70 keV

**Table 3:** Performances of DECT quantitative parameters for the differentiation of benign and malignant breast lesions

Variable	AUC	Threshold	Sensitivity (%)	Specificity (%)	Accuracy (%)
Conventional attenuation (HU)					
In the arterial phase	0.64	48.25	56.3	69.0	58.7
In the venous phase	0.68	71.05	76.2	58.6	72.9
40-keV VMIs attenuation (HU)					
In the arterial phase	0.62	56.65	71.4	55.2	68.4
In the venous phase	0.66	85.38	97.6	34.5	85.8
IC (mg/mL)					
In the venous phase	0.65	1.02	78.6	51.7	73.5
nIC (ratio of lesion and normal parenchyma)					
In the venous phase	0.67	7.03	73.8	62.1	71.6
Zeff					
In the venous phase	0.66	7.87	81.0	51.7	75.5
nZeff (ratio of lesion and normal parenchyma)					
In the venous phase	0.68	1.08	81.0	58.6	76.8
λ_HU40-70 keV_ (HU/keV)					
In the venous phase	0.65	1.93	81.0	51.7	75.5

All *p* > 0.05 in the Delong test

### DECT-based model construction and evaluation

**Fig. 2:**
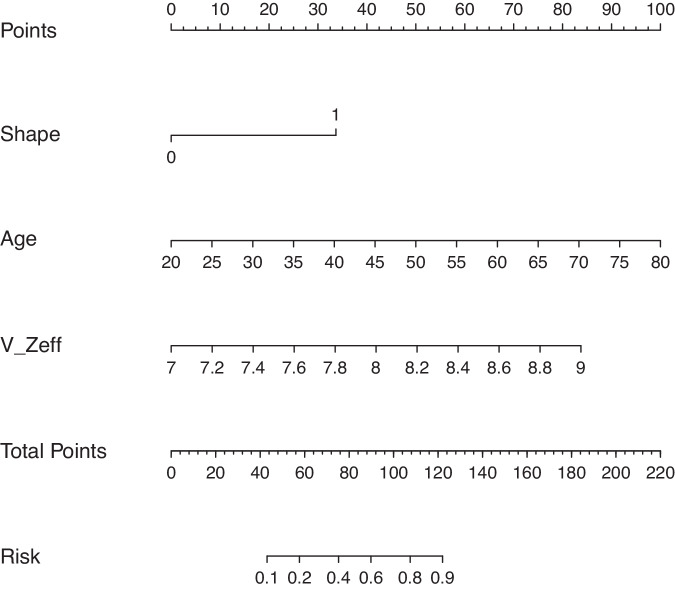
Visualized nomogram of the model. The effective atomic number quantification-based model was developed in the training cohort, with lesion shape, patient age, and effective atomic number in the venous phase (V_Zeff) included. Lesion shape was divided into two groups, with 0 denoting regular shape and 1 denoting irregular shape

The clinical characteristics, DECT-based morphological features, and DECT quantitative parameters with statistical significance in univariate analysis were further included in multivariate logistic regression analysis, which revealed age, lesion shape, and Zeff in the venous phase as independent predictors (Table 4). For individualized prediction of breast lesions, a DECT-based model was constructed by incorporating these predictors and the corresponding regression coefficients and was visualized by the nomogram (Fig. 2).

**Table 4:** Multivariate logistic regression analysis of the DECT-based model

Variable	β	Adjusted OR	95%CI	*p*
Intercept	−22.778			
Shape	1.875	6.518	2.360–18.002	< 0.001
Age	0.093	1.097	1.045–1.152	< 0.001
V_Zeff	2.332	10.3	2.414–43.954	0.002

**Fig. 3:**
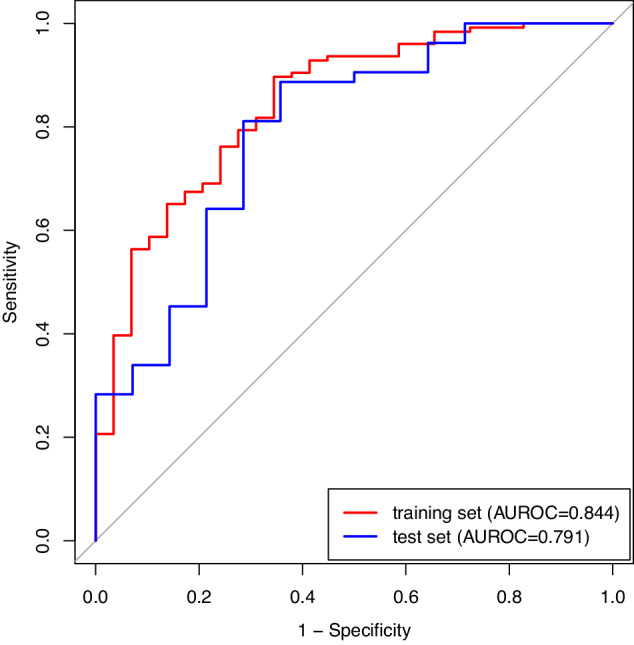
ROC curve analysis showing the diagnostic abilities of the DECT-based model in the training and test cohorts. No significant difference was found in AUC between the two cohorts (DeLong test, *p* = 0.53)

**Fig. 4:**
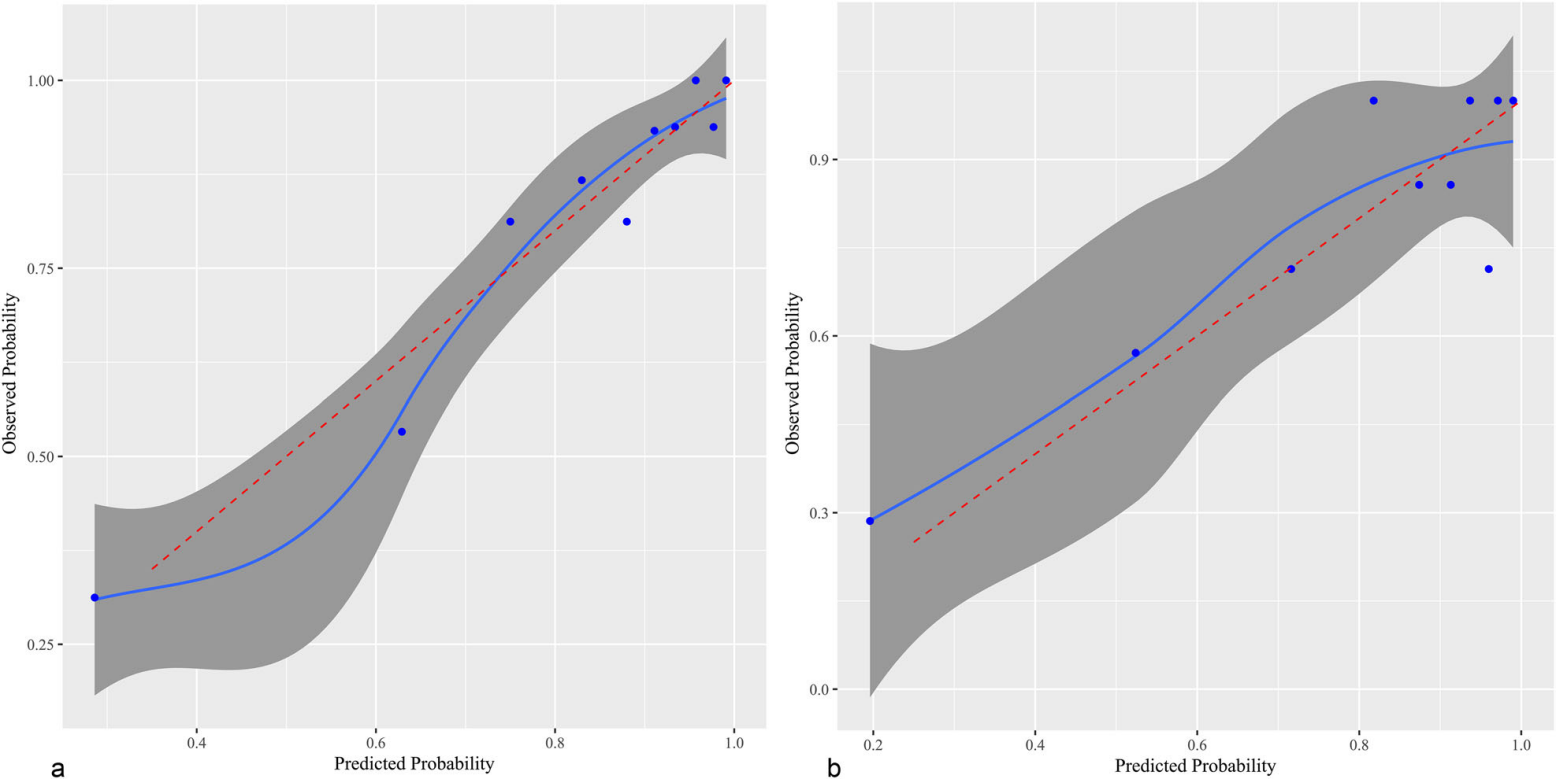
Agreement between the observed frequency and model-predicted probability of benign and malignant breast lesions in the training and test cohorts. Calibration curves of the model for the training (a) and test (b) cohorts. The solid blue line denotes the predictive performance, and the gray-shaded area denotes the 95% CI. Close fitting of the predictive performance to perfect prediction (dashed red line) indicates the model was well fitted. The Hosmer-Lemeshow test showed *p*-values of 0.85 and 0.33 in the training and test cohorts, respectively. Circles denote different data points

**Fig. 5:**
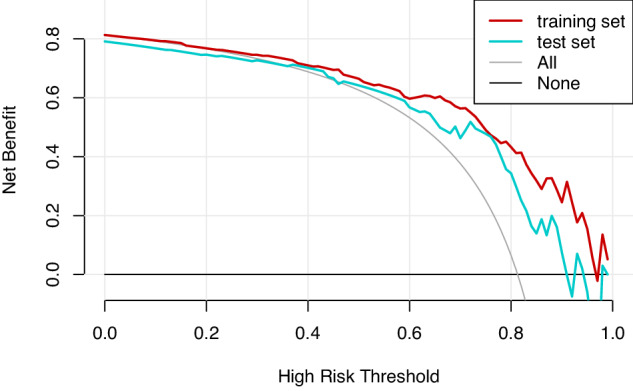
Decision curve analysis in the training and test cohorts. The net benefit of using the model for clinical decision-making exceeded that of applying default schemes to treat all patients (treat all scheme) or treat no patients (treat none scheme) within a wide range of threshold probabilities in both cohorts

**Fig. 6:**
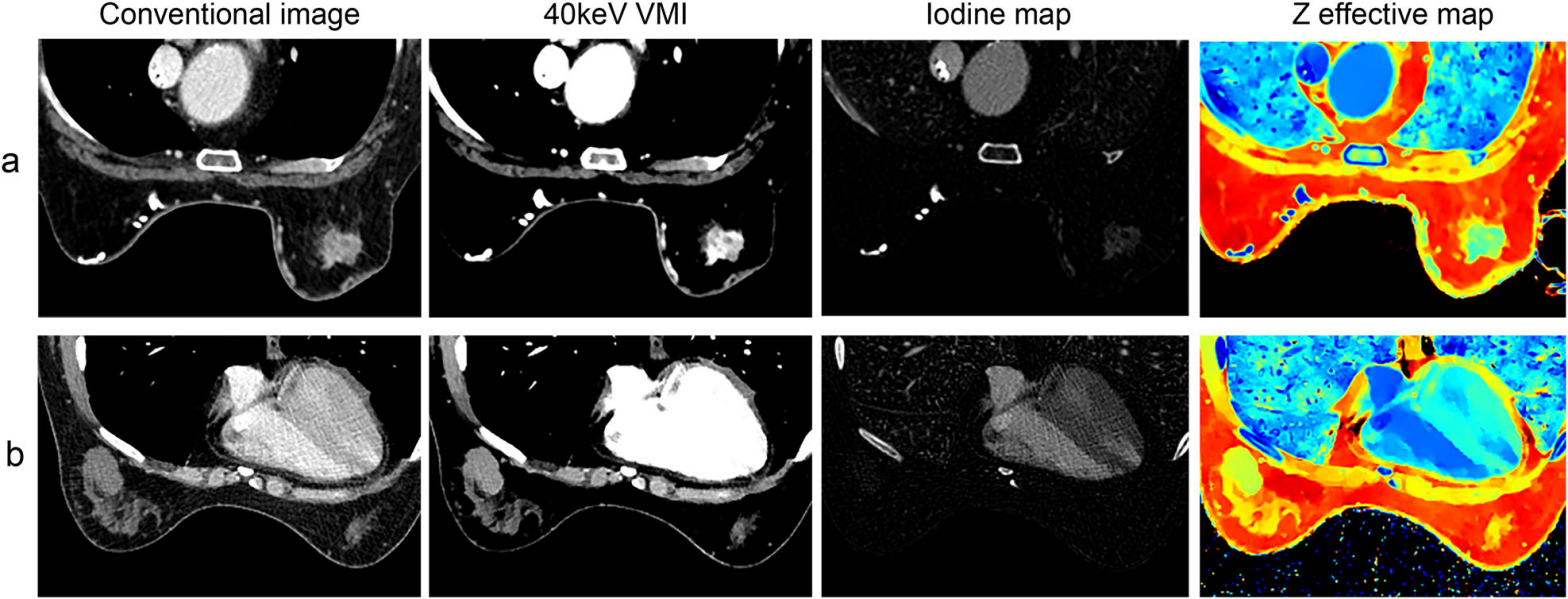
Examples of application of model to predict probability of malignant breast lesions. **a** 68-year-old woman with left invasive breast cancer. Conventional image and 40 keV virtual monoenergetic image (VMI) show that lesion was irregular, and the effective atomic number on Z effective map was 8.08. Model revealed that probability of patient having malignant breast lesion was 0.986. **b** 46-year-old woman with right breast phyllodes tumor. Conventional image and 40 keV VMI show that lesion was irregular, and the effective atomic number on Z effective map was 7.56. Model revealed that probability of patient having malignant breast lesion was 0.294

The model showed promising diagnostic performance in distinguishing between benign and malignant breast lesions, with AUCs of 0.844 (95%CI, 0.764–0.925) and 0.791 (95%CI, 0.647–0.935) in training and test cohorts, respectively (Fig. 3). The DeLong test detected no significant difference in AUC between the two cohorts (*p* = 0.53). The diagnostic performances of the model in the training and test cohorts are shown in Table 5. Using the optimal threshold of 0.705, the sensitivity, specificity, and accuracy of the model were 89.7%, 65.5%, and 85.2% in the training cohort, respectively, versus 86.8%, 64.3%, and 82.1% in the test cohort, respectively. And corresponding false negative rates were 10.3% and 13.2% in the training and test cohorts, respectively. Moreover, Fig. 4 shows the calibration analysis, which suggested a good concordance between the model-predicted probability and actual frequency in both cohorts. The Hosmer-Lemeshow test yielded *p*-values of 0.85 and 0.33 in the training and test cohorts, respectively. The decision curve analysis demonstrated that the novel model provided enhanced net benefits over the default simple strategies within a certain range of threshold (training cohort, 25–98%; test cohort, 45–90%) in both cohorts (Fig. 5). In addition, two examples of the application of the model to predict the probability of malignant breast lesions detected on DECT are illustrated in Fig. 6.

**Table 5:** Diagnostic performances of the model in the training and test cohorts

	AUC	95% CI	Sensitivity (%)	Specificity (%)	Accuracy (%)
Training cohort	0.844	(0.764–0.925)	89.7	65.5	85.2
Test cohort	0.791	(0.647–0.935)	86.8	64.3	82.1

*CI* confidence interval

## Discussion

In this study, DECT quantitative parameters were higher for malignant breast lesions compared with benign lesions. The multivariate regression analysis revealed that age, lesion shape, and Zeff in the venous phase were independent factors for differentiating between benign and malignant breast lesions detected on DECT. Furthermore, a DECT-based model was constructed by integrating these predictors. The AUC, sensitivity, specificity, accuracy, and false negative rates were 0.844, 89.7%, 65.5%, 85.2%, and 10.3% in the training cohort and 0.791, 86.8%, 64.3%, 82.1%, and 13.2% in the test cohort, respectively.

This study demonstrated that attenuation on conventional images and 40-keV VMIs, IC, Zeff, and λ_HU_ were higher in malignant breast lesions compared with benign ones, corroborating previous studies [17, 18]. This might imply that malignant breast lesions have more potential microvessels and angiogenesis, as these DECT quantitative parameters correlate to the distribution and content of contrast agents in lesions. Several studies [36–38] revealed DECT quantitative parameters in the venous phase have better diagnostic abilities compared with counterparts in the arterial phase. This work also found there were more DECT quantitative parameters with statistically significant differences in the venous phase compared with the arterial phase. Future studies focusing on breast lesions could potentially apply venous phase scans only to reduce radiation exposure. A possible explanation was that the DECT protocol applied in this study was based on a chest-enhanced CT protocol, which might not have allowed the contrast agent to adequately penetrate breast lesions in the arterial phase, making venous phase scans more informative for the assessment of underlying microvessels within the lesions.

In a previous study [28], patients with malignant breast lesions were aged 50 to 54 years, which was consistent with the present study, and age was further proven as an independent factor. For breast lesions incidentally detected on CT, irregular margins, irregular shape, and rim enhancement were highly predictive of malignant breast lesions [30, 39]. The current findings also confirmed that breast lesions with non-circumscribed margins and irregular shapes were more common in malignant lesions, but inner enhancement of the lesions showed no significant difference in this study. This might be because heterogeneous enhancement was not subdivided into clumped or clustered ring enhancement and rim enhancement, thereby concealing potential differences. To minimize individual variations in circulation, the IC and Zeff of lesions were divided by those of the aorta or normal breast parenchyma, respectively, as previously proposed [16, 17]. However, it was worth noting that Zeff in the venous phase was an independent predictor, instead of the normalized parameters. Zeff is a measure of material composition that reflects the atomic number of the element showing the same x-ray attenuation coefficient. Malignant breast lesions typically exhibit increased angiogenesis [40]. With more angiogenesis occurring in breast lesions, the more the iodine would present, and the higher the effective atomic number would be. However, with excessive amounts of iodine contrast in the aorta, it can be difficult to distinguish a small difference between the nIC_lesion/aorta_ and nZeff_lesion/aorta_ for benign and malignant breast lesions. Therefore, further research is required to determine the practical value of the standardization approach involving the division of IC or Zeff of lesions by that of the aorta.

In contrast to prior work [18], the constructed model consisted of clinical and morphological features and a quantitative parameter. The model demonstrated promising discriminative power in both cohorts, confirming its robustness. The calibration curves and the decision curve analysis also showed that the model provided a good fit and a greater net benefit within a certain threshold range compared with the default simple strategies in both the training and test cohorts. To the best of our knowledge, this study first combined clinical and morphological characteristics with DECT quantitative parameters for the identification of breast lesions detected on DECT. While the lesions included in this study were not incidental breast lesions on CT, the findings could be useful for differentiating benign and malignant breast lesions incidentally detected on DECT.

This study had several limitations. Firstly, it was a single-center study with an imbalance in the numbers of benign and malignant breast lesions, which could introduce bias. Secondly, some invisible breast lesions on DECT images were excluded, and most of them were subsequently identified as benign lesions. Consequently, the difference in conspicuity between benignity and malignancy on DECT images may be overlooked. Thirdly, the consensus reached in morphological feature analysis may not represent the individual radiologists with different levels of experience. Fourthly, the performance of the model in different types of breast cancers was not explored, considering the limited number of some types of breast cancers. In addition, it is possible that other new DECT parameters could further improve the differential diagnostic performance for breast lesions. Future multicenter studies including a more balanced dataset and additional DECT quantitative parameters, such as electron density are required. Furthermore, validation procedures are warranted to assess the model’s performance, particularly in the context of incidental breast lesions.

In conclusion, a DECT-based model, integrating age, lesion shape, and Zeff in the venous phase, had favorable diagnostic performance and can be beneficial for the identification of benign and malignant breast lesions detected on DECT to assist general radiologists in deciding further work-up.

### Supplementary information

ELECTRONIC SUPPLEMENTARY MATERIAL

## Data Availability

The data used during this study are available from the corresponding author on reasonable request.

## Abbreviations

AUC: Area under the curve
BPE: Background parenchymal enhancement
CI: Confidence interval
CTDIvol: CT dose index volume
DECT: Dual-energy CT
DLP: Dose length product
FGT: Fibroglandular tissue
HU: Hounsfield unit
IC: Iodine concentration
nIC: Normalized iodine concentration
NME: Non-mass enhancement
nZeff: Normalized effective atomic number
ROC: Receiver operating characteristic
ROI: Regions of interest
VMI: Virtual monoenergetic image
Zeff: Effective atomic number
λ_HU_: Slope of the curve
