# Supplementary material for: Differentiating between benign and malignant breast lesions using dual-energy CT-based model: development and validation
Source: Insights Imaging. 2024 Jul 10;15:173. doi: 10.1186/s13244-024-01752-2 (PMC11233492; doi:10.1186/s13244-024-01752-2)
Supplement: Supplementary file 1 — ELECTRONIC SUPPLEMENTARY MATERIAL [file 13244_2024_1752_MOESM1_ESM.pdf]

Differentiating between benign and malignant breast lesions using dual-energy CT-based model: development and validation

ELECTRONIC SUPPLEMENTARY MATERIAL

Table E1. Dual-energy CT Scan Parameter

Scan Parameters	
Tube voltage, kVp	120
Tube current, mA	automated exposure control modulation
Pitch factor	0.609
Rotation time, s	0.5
Collimation, mm	64×0.625
Slice thickness, mm	1
Slice increment, mm	1

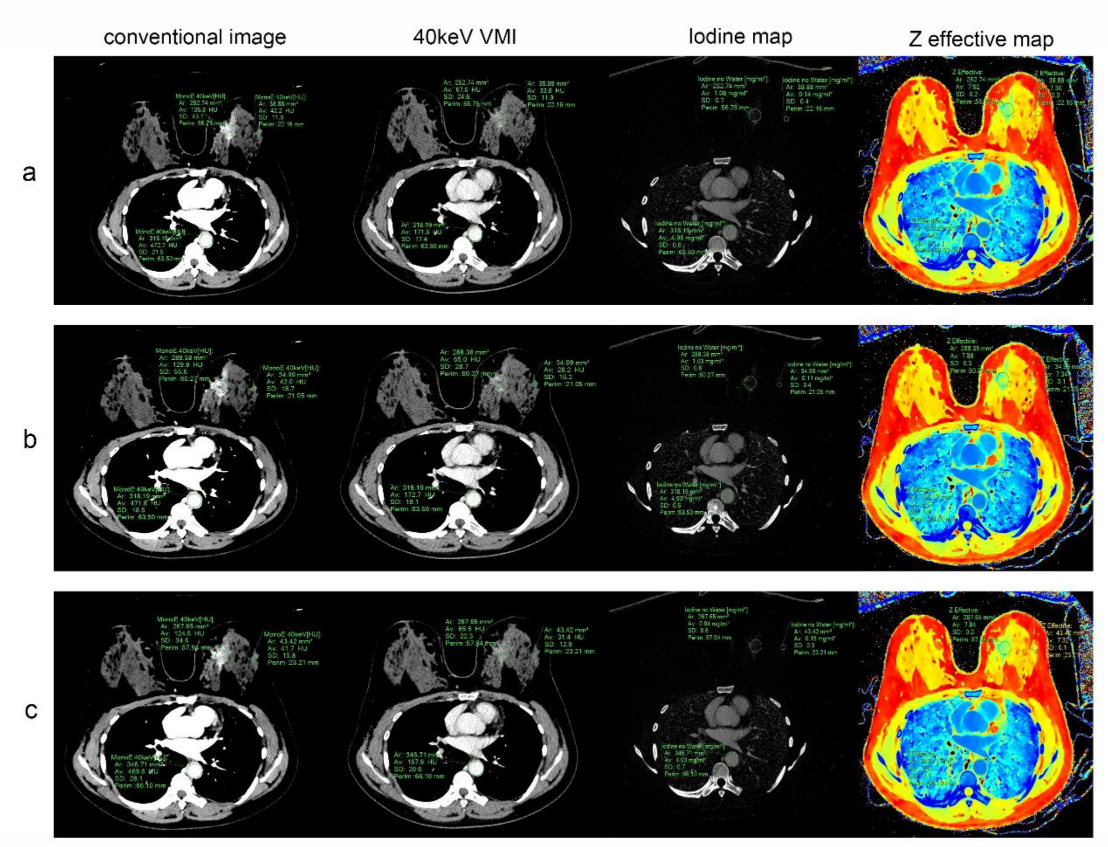

Figure E1. An example of ROI placement. In the venous phase, ROIs were placed as large as possible on the breast lesion, the normal breast parenchyma, and the aorta in the axial plane showing the maximum dimension of a breast lesion (a) plus its adjacent upper (b) and lower slices (c). Measurements on three slices were then averaged.

Table E2. Pathological findings in training and test cohort.

Pathological findings	Training cohort (%)	Test cohort (%)
Malignant	126 (100)	53 (100)
Invasive breast cancer	104 (82.5)	49 (92.4)
DCIS	13 (10.3)	2 (3.8)
Papillary carcinoma	5 (4.0)	0 (0)
Others	4 (3.2)	2 (3.8)
Benign	29 (100)	14 (100)
Fibroadenoma	11 (37.9)	8 (57.1)
Intraductal papilloma	6 (20.7)	4 (28.6)
Phyllodes tumor	4 (13.8)	2 (14.3)
Mastopathy	3 (10.3)	0 (0)
Others	5 (17.3)	0 (0)

Note: Variables were expressed as frequency and frequency distribution. DCIS, ductal carcinoma in situ.
